# Low Noise and Drift Reconfigurable Solution‐Processed Chalcogenide Phase Change Metasurfaces

**DOI:** 10.1002/smtd.202501088

**Published:** 2025-07-24

**Authors:** Mahirah Zaini, Abbas Sheikh Ansari, Joshua Perkins, Avik Mandal, Yedeng Fei, Ahmed H. Elfarash, Tony Kong, Behrad Gholipour

**Affiliations:** ^1^ Nanoscale Optics Lab, Electrical and Computer Engineering Dept University of Alberta Edmonton AB T6G 2R3 Canada

**Keywords:** metamaterials, phase change, reconfigurable, solution‐processed, spin coat, thermo‐optic

## Abstract

Chalcogenide glasses are increasingly favoured as the programmable layer of choice in reconfigurable optoelectronic platforms, enabling diverse signal modulation, display, and memory device applications over the past decade. These applications capitalize on the amorphous‐to‐crystalline phase transition of these alloys, often produced using expensive ultrahigh‐vacuum physical vapor deposition (PVD) methods. Here, a cost‐effective, solution‐processed approach is presented to synthesizing chalcogenide phase change materials (PCMs). Our results show that optical‐grade antimony sulfide (Sb_2_S_3_) can be deposited onto various substrates at subwavelength thicknesses. Notably, these films demonstrate non‐volatile phase change modulation contrasts comparable to PVD methods, with significantly lower volatile thermo‐optic response, promising enhanced performance by reducing noise and drift. The first reconfigurable phase change chalcogenide metasurface formed from solution‐processed PCM films are also introduced, which can be patterned into polarization‐sensitive subwavelength nanograting metasurfaces without degradation, allowing for period‐dependent resonances and large modulation contrasts. The liquid nature of the deposition technique is perfectly suited for inclusion in display technologies and the integration of various emitters and active nanoparticles into PCM films, paving the way for new hybrid PCM composites, offering numerous solutions for emerging quantum and neuromorphic photonic platforms while lowering production costs.

## Graphical abstract

Chalcogenide glasses, favored in reconfigurable optoelectronics, rely on costly ultra‐high vacuum‐based PVD methods. A cost‐effective, solution‐based approach to synthesize these materials, producing high‐quality antimony sulfide films with comparable phase change performance are introduced. This enables innovative metasurfaces and hybrid composites for advanced quantum and neuromorphic photonics, reducing costs and expanding applications.

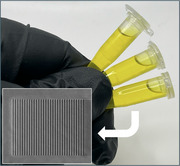

## 1 Introduction

Chalcogenide phase‐change materials (PCMs) have emerged as a groundbreaking platform in engineering, revolutionizing numerous fields such as data storage, telecommunications, and cutting‐edge computing technologies. These alloys, which are based on group‐16 ‘chalcogen’ elements (sulfur, selenium, and tellurium) covalently bound to ‘network formers’ such as arsenic, germanium, antimony, indium, or gallium, present a variety of technologically useful properties. These include high refractive index and plasmonic properties, infrared (IR) transparency, and high levels of optical nonlinearity. Most notably, they offer the unique ability to switch in a non‐volatile manner between amorphous and crystalline phases, displaying markedly different optoelectronic properties. This transformative switching can be triggered through thermal, electronic, or optical stimuli across a wide variety of compositions and stoichiometries. Each variation boasts distinct advantages tailored to specific applications, making PCMs essential in advancing a variety of technological landscapes.

The amorphous‐to‐crystalline phase transition, which is the basis for the non‐volatile memory functionality most exploited in these semiconductors, is an annealing process that may be initiated globally or locally by furnace, laser‐ or electrical current‐induced heating to a temperature above the material's glass‐transition point *T_g_* but below its melting point *T_m_*.^[^ ^1^ ^]^ The reverse transition (a melt‐quenching process) can be driven by shorter, higher‐energy pulsed excitations that bring the material briefly to a temperature above *T_m_*.^[^ ^2^ ^]^ This reversible transition brings about large optical contrasts (typically Δn > 1) over a broad wavelength range and reversible switching between amorphous and crystalline states with high cyclability and switching speeds down to sub‐nanosecond timescales. With these advantages, reconfigurable photonic platforms employing phase change materials are presenting a promising route to low‐footprint optical components that incorporate memory functionality.^[^ ^3^, ^4^ ^]^ Notably, these components also promise reduced susceptibility to extrinsic perturbations, such as noise, thermal/electrical drift, and crosstalk, when compared to traditional reconfigurable platforms that utilize volatile thermo‐optic and carrier modulation phenomena.

The distinct switching capability inherent to phase change materials (PCMs) is significantly augmented by their high refractive indices and diverse nonlinear optical properties across industrially relevant consumer and telecommunication bands. Recently, PCMs have become crucial in advancing free‐space platforms such as active plasmonic and photonic metamaterial technologies, which incorporate built‐in memory for enhanced adaptive operation. This innovation has facilitated an array of switchable, tunable, and reconfigurable optical functionalities, either in an all‐chalcogenide architecture or in conjunction with plasmonic metal nanostructures.^[^ ^3^, ^5^, ^6^, ^7^, ^8^, ^9^, ^10^, ^11^, ^12^, ^13^ ^]^ These advancements effectively address a spectrum of applications, including electro‐optical and all‐optical signal switching, polarization modulation, beam steering, and adaptive multispectral imaging.^[^ ^14^, ^15^, ^16^, ^17^, ^18^, ^19^, ^20^, ^21^, ^22^ ^]^ Furthermore, as these alloys offer large reconfigurable non‐volatile modulation contrasts, for operation across telecommunications bands, very recently, alloys such as antimony sulfide (Sb_2_S_3_) have emerged as a lower‐loss choice to their telluride counterparts for next‐generation silicon‐centric integrated photonic architectures.^[^ ^23^ ^]^ Their application spans micro‐ring resonator‐based switches,^[^ ^17^ ^]^ on‐chip photonic memory,^[^ ^24^, ^25^ ^]^ all‐optical 1 × 2 switches,^[^ ^21^ ^]^ nanogap integrated switches,^[^ ^26^ ^]^ programmable phase shifters,^[^ ^27^ ^]^ photonic synapses,^[^ ^28^, ^29^ ^]^ and cascaded self‐learning networks,^[^ ^30^ ^]^ as well as fiber‐integrated switches.^[^ ^31^, ^32^ ^]^ Beyond their non‐volatile phase change properties, PCM's also exhibit broadband switchable, volatile, thermo‐optic properties that manifest themselves as broadband temperature‐dependent dispersive changes in refractive index and extinction coefficient across both amorphous and crystalline phases, pointing to their promise for use as both the volatile and non‐volatile layer in emerging signal modulation architectures.

Despite these remarkable properties and the global research fervor surrounding this family of materials, the high manufacturing cost is one of the biggest barriers to the widespread integration of such materials in all aspects of industrial optoelectronics. These alloys are generally grown on substrates using ultra‐high vacuum physical and chemical vapor deposition (PVD/CVD) techniques. The use of ultra‐high vacuum physical vapor deposition (PVD/CVD) techniques, which necessitate expensive cleanroom environments, dramatically increases production expenses and serves as a barrier to the introduction of these materials into many processes. Here, we directly address this obstacle by utilizing wet‐chemistry‐based solution‐processed techniques to enable the deposition of phase change films on diverse substrate types. This eliminates the need for cleanroom‐based ultrahigh vacuum techniques for the growth of nanoscale thickness optical‐grade phase change materials.

## 2 Results and Discussion

**Figure 1:**
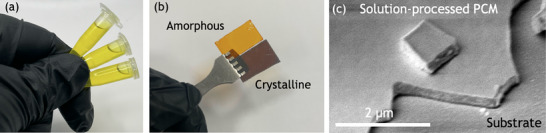
Optical‐grade solution‐processed phase change materials. a) Camera image of solution in vials prior to spin coating b) Typical 380nm thick spin‐coated Sb_2_S_3_ samples on glass substrates in as‐deposited amorphous and annealed/crystalline phases, as labelled. c) Scanning electron microscope of a 380 nm thick sample on a glass substrate.

**Figure 2:**
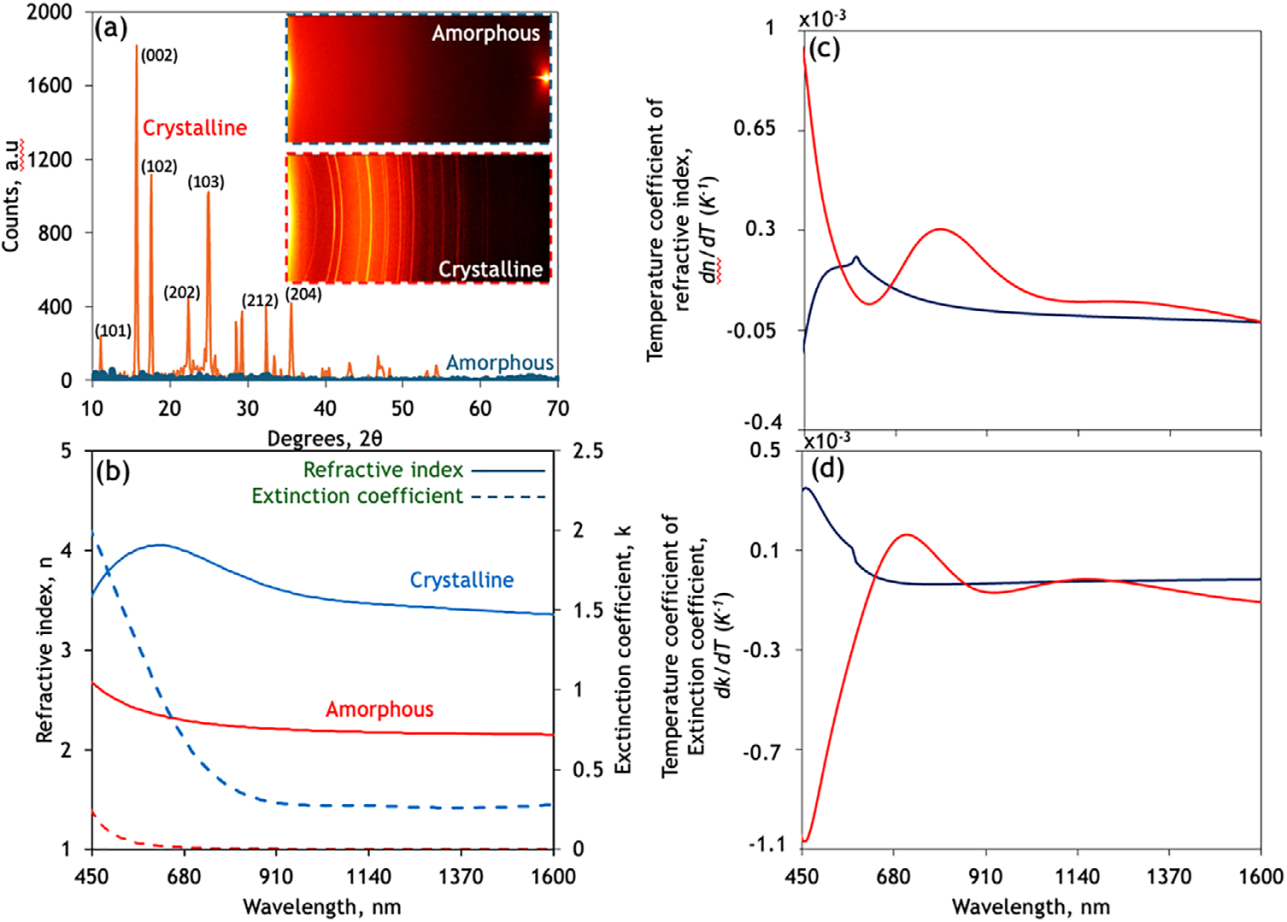
Volatile and non‐volatile reconfigurable properties of solution‐processed phase change materials. a) X‐ray diffraction of 380nm thick as‐deposited amorphous and annealed/crystalline film on glass substrate b) Spectral dispersion of refractive index (n) and extinction‐coefficient (k) of a typical solution‐processed Sb_2_S_3_ film in both amorphous and crystalline phases measured through variable angle spectroscopic ellipsometry. c) and d) Spectra dispersion of temperature coefficient of (c) refractive index (*d*n/*d*t) and (d) extinction coefficient (*d*k/*d*t) across amorphous and crystalline phases of the PCM alloy.

In our experiments, solution‐processed films of antimony sulfide (Sb_2_S_3_) across a range of thicknesses were synthesized using wet chemistry techniques and deposited uniformly onto silicon and glass substrates (**Figure** **1**). Prior to the deposition, the phase change solution was made by mixing 1500 µL carbon disulfide (CS_2_), 2000 µL ethanol, and 0.291 grams of antimony oxide in a 20 mL vial using a magnetic stirrer. A 2000 µL n‐butylamine is slowly injected into the vial and left to stir overnight to dissolve the oxide and achieve a clear solution. To deposit a target thickness of ≈380 nm for example, we diluted the mixed solution with ethanol using 2:1 (phase change solution: ethanol) ratio. The diluted solution is then spin‐coated onto silicon and glass substrates at 5000 rotations per minute (RPM) for 40 s. Next, the sample is soft baked in a vacuum furnace at 100°C for 5 min to achieve the as‐deposited amorphous phase. The thicknesses were measured using a profilometer (Alpha‐Step IQ), and phase transitions were instigated by annealing in a furnace under vacuum conditions at 300°C. X‐ray diffraction (XRD) (Bruker D8D plus XRD‐SAXS) was used to confirm crystallization to an orthorhombic‐dominated phase as expected from this alloy,^[^ ^33^ ^]^ and Energy dispersive X‐ray (EDX) (Oxford X‐Max 50mm configured to Zeiss EVO MA10 SEM) was utilized to confirm the chemical composition of the as‐deposited/amorphous and annealed/crystallized films to be stable at Sb_2_S_3_ (**Figure** **2**a and S1, Supporting Information). Optical properties were measured in both amorphous and crystalline states across the wavelength band 400 nm ≥ λ ≤ 1600 nm using an ellipsometer (Woollam VASE spectroscopic) fitted with a Linkam heating stage (model: LTS420E‐PB4) focusing on the temperature range 30 to 80°C in 10°C intervals. All measurements were carried out across three angles (65°, 70° and 75°), and both forward (heating) and backward (cooling) temperature sweeps were conducted to discern any potential temperature hysteresis.

Figure 2 illustrates the switchable wavelength dependence of both the refractive index and extinction coefficient for the fabricated solution‐processed PCMs. The as‐deposited amorphous films exhibit a high‐refractive index and low optical loss behaviour, demonstrating a refractive index as high as n > 4 at λ <800 nm that decays to n ≈ 2 at λ = 1550nm and corresponding extinction coefficient, k ≈ 0.5 × 10^−4^ across much of the telecommunication band (Figure 2b). The non‐volatile phase transition brings about a stark change in static optical properties with an average change of n ≈ 1.2 across much of the near‐infrared frequencies, including all telecommunication bands. This is accompanied by an increase in optical loss on the order of k ≈ 0.26 across the aforementioned bands. Notably, the non‐volatile change observed in these solution‐processed films is comparable to that demonstrated in antimony sulfide films made through costly PVD growth techniques.^[^ ^19^ ^]^ The refractive indices (n) for crystalline and amorphous Sb₂S₃ presented exhibit peak values of ≈4 and 2.7, respectively, at shorter wavelengths (λ ≈ 450 nm), stabilizing at n ≈ 3.5 (crystalline) and 2.5 (amorphous) for longer wavelengths (1000 < λ < 1600 nm). Literature values for Sb₂S₃ films fabricated via physical vapor deposition (PVD) generally report similar peak refractive indices ranging from 3.8 to 4.4 (crystalline) and 2.6 to 3.2 (amorphous) in the visible‐to‐near‐IR range, demonstrating that the current solution‐processed films closely match established benchmarks (Ref. 19).

The extinction coefficients observed for solution‐processed films are ≈2.0 to 2.2 (crystalline) and 0.3 to 0.5 (amorphous) at shorter wavelengths (450 < λ < 680 nm), and decrease significantly toward near‐infrared wavelengths, reaching nearly zero for amorphous films beyond λ = 900 nm. These values are comparable or superior to typical values reported for PVD‐grown films, which range approximately between 1.5 to 2.5 (crystalline) and 0.2 to 0.8 (amorphous) in the visible spectrum (19, 20).

Additionally, the solution‐processed films presented here achieve an optical modulation depth defined as the difference in refractive indices between crystalline and amorphous phases of approximately Δ*n* ≈ 1.2 at visible wavelengths (450 < λ < 700 nm), comparable to the best values (Δ*n* ≈1.0–1.4) reported for PVD‐grown films in the literature (19). This quantitative parity underscores the suitability of solution‐based processing for scalable manufacturing, thus enhancing the industrial relevance of our approach.

Therefore, the solution‐processed Sb₂S₃ films exhibit optical properties that are quantitatively comparable and, in some cases, superior to those fabricated by conventional PVD techniques (See Table S2, Supporting Information).

Figure 2c and d illustrate the switchable wavelength dependence of both the refractive index (*d*n/*d*t) and extinction coefficient (*d*k/*d*t) components of the volatile thermo‐optic properties measured for the solution‐processed films in both amorphous and crystalline phases. Uniquely, the sign of both components switches across high‐energy visible frequencies (λ < 500nm) upon a phase transition and both the magnitude and switching contrast are minimized across telecommunication frequencies. The observed values for solution‐processed Sb_2_S_3_ in amorphous and crystalline phases are 2 × 10^−5^ and −5.4 × 10^−6,^ respectively at λ = 1550nm. Most notably, these values are ten times lower than their PVD counterparts reported in the literature.^[^ ^34^ ^]^ This points to solution‐processed PCMs being an ultra‐stable programmable layer with less susceptibility to environmental drifts and supply‐related noise, even when compared to PCM films made through PVD techniques.

To demonstrate the potential of solution‐processed chalcogenide films as a low optical loss platform for nanophotonic applications, amenable to typical patterning techniques, we show that such solution‐processed films can be used to realize reconfigurable metasurfaces consisting of sub‐wavelength nanograting arrays. We show that these metasurfaces can be fabricated using focused ion beam (FIB) techniques with high finesse, presenting structurally tunable resonances that can be reconfigured upon a non‐volatile phase transition.

**Figure 3:**
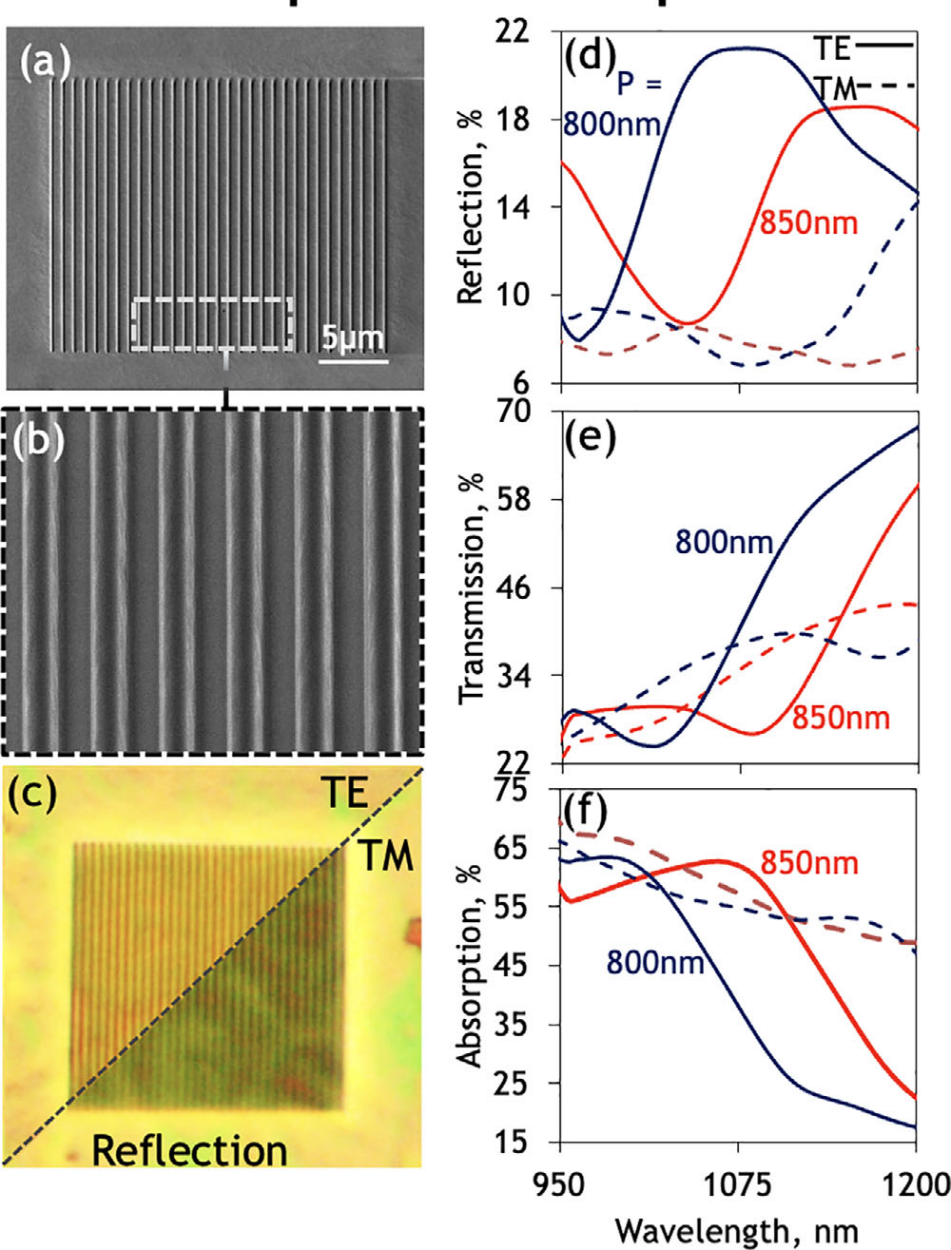
Solution‐processed phase change chalcogenide metasurface. a, b) Scanning electron microscopy of metasurface alongside c) Reflection optical microscopy of an individual nanograting metasurface when illuminated with light in both TE and TM polarization as labelled. d–f) Microspectrophotometry of as‐deposited solution‐processed nanograting metasurfaces showing TE‐polarized (solid) d) Reflection, e) transmission, and (f) absorption for a selection of periods P = 800 – 850nm, overlaid with the equivalent response, under TM‐polarised illumination (dashed).

Our work employs non‐diffracting, sub‐wavelength nanograting^[^ ^35^, ^36^, ^37^ ^]^ metasurface patterns having periods, *P*, in the 800–850 nm range, fabricated by etching spin‐coated as‐deposited amorphous films on glass substrates (**Figure** **3**a,b). Standalone, optically thick solution‐processed Sb_2_S_3_ is a high‐index, transmissive medium (Figure S3, Supporting Information) with a reflectivity that is substantially modified in this spectral range by the subwavelength nanograting structures.

**Figure 4:**
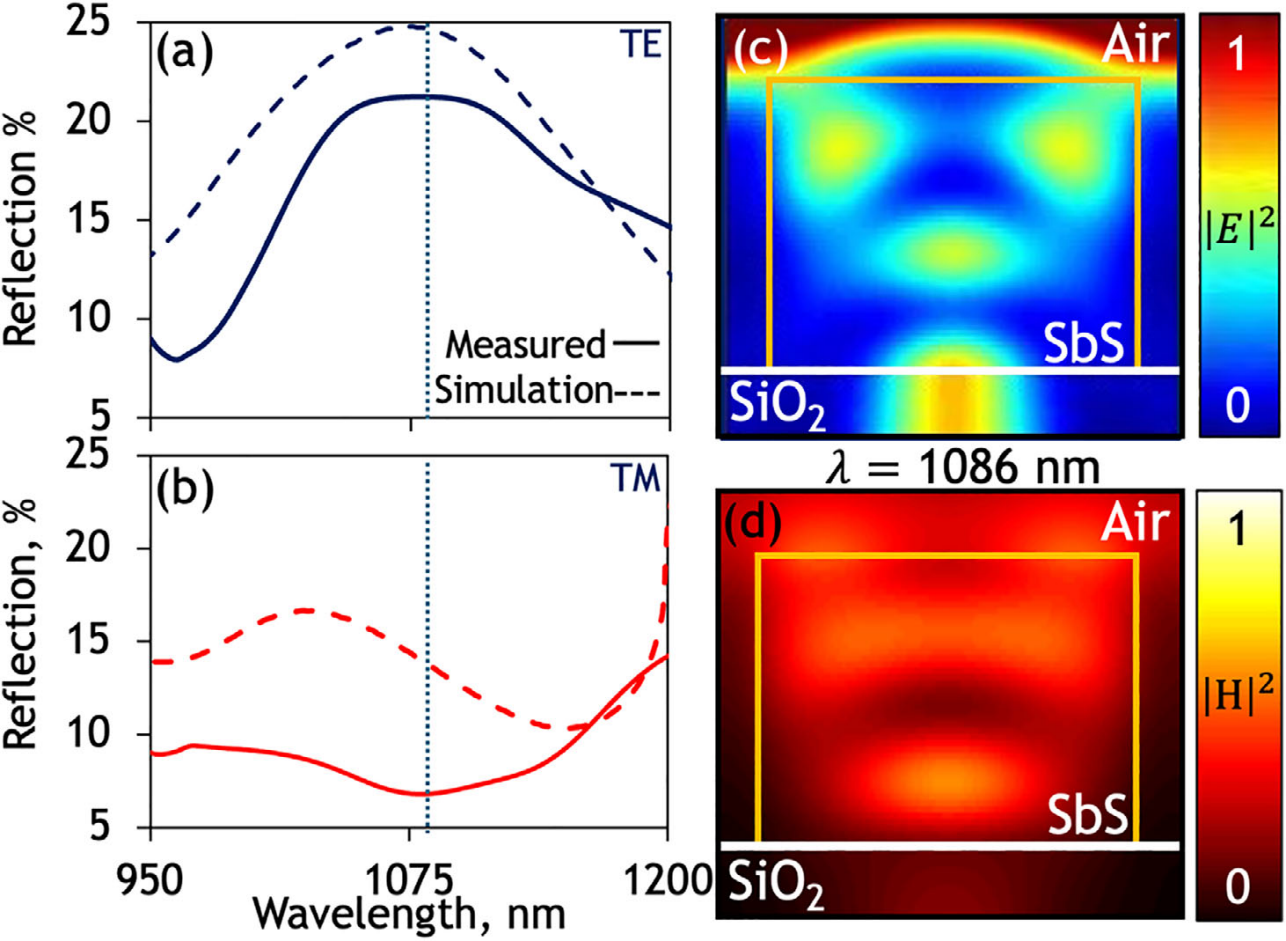
Numerical simulations of the optical response of solution‐processed phase change chalcogenide metasurface. a) Microspectrophotometry of as‐deposited solution‐processed nanograting metasurfaces showing TE‐polarized a) Reflection and b) transmission, for a metasurface with period P = 800 nm, overlaid with the equivalent simulated response (dashed). Corresponding Distributions of the normalized c) electric and d) magnetic fields for a unit cell of a P = 800 nm nanograting at λ = 1086 nm.

Patterning such a structure with subwavelength nanograting metasurfaces produces reflection resonances which vary as a function of the nanograting period for incident light polarised parallel (TE) to the grating lines due to the high‐index nature of this PCM across this spectral range. Due to the highly anisotropic nature of nanograting resonators, the optical response depends on the polarization of incident light (Figure 3d–f). As such, the observed response of nanograting metasurfaces is highly sensitive to polarisation, with resonant response disappearing with incident polarization perpendicular to the grooves of the grating (TM). In this orientation, the nanograting behaves as a linear medium with a non‐dispersive effective refractive index related to that of the PCM‐substrate stack and the fill fraction within the grating structure. The observed optical response is elegantly reproduced in finite difference time domain simulations (**Figure** **4**a,b). The model (in Ansys Lumerical) assumes a lossless non‐dispersive refractive index of 1.46 for the silica substrate, normally incident narrowband plane wave illumination and, by virtue of periodic boundary conditions, a grating pattern of infinite extent in the substrate plane. Discrepancies between experiment and numerical simulations are due to manufacturing imperfections, such as deviations from the ideal model geometry e.g slight over‐milling of grating lines into the substrate, slanted side walls, and to contamination and stoichiometric change in the PCM during milling, which may create changes in refractive index as has also been reported with PVD films.

As amorphous solution‐processed Sb_2_S_3_ behaves as a dielectric, the electric and magnetic field distribution at the resonant frequencies is confined inside the nanostructured dielectric medium due to the relatively high refractive index (Figure 4c,d). This enables the coupling of incident light to guided and diffracted modes within the structure, leading to the constructive interference of light trapped inside the PCM layer, akin to whispering‐gallery or leaky‐mode resonances found in larger square and micro‐disk resonators.^[^ ^38^, ^39^ ^]^

By utilizing the phase‐change properties of Sb_2_S_3_, the resonances of these solution‐processed all‐chalcogenide metasurfaces can be optically switched in a non‐volatile manner. The resulting change in the dielectric function of the PCM leads to a variation in the spectral dispersion of the nanograting resonances, bringing about substantial changes in the metasurface transmission and reflection (Figure S4, Supporting Information).

**Figure 5:**
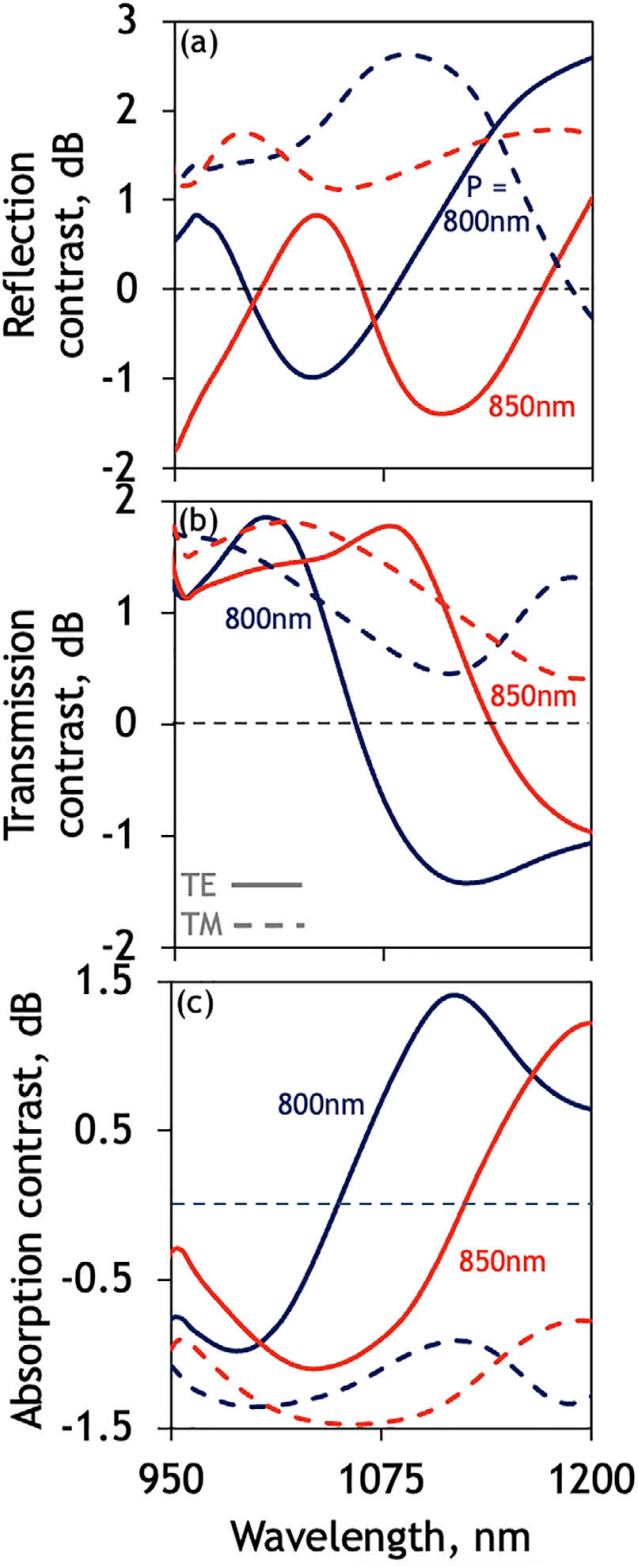
Non‐volatile reconfiguration of solution‐processed phase change metasurfaces. a) Microspectrophotometry of solution‐processed nanograting metasurfaces showing the observed modulation contrast when illuminated with TE‐polarized (solid) a) reflection, b) transmission, and c) absorption for a selection of periods P = 800–850nm, overlaid with the equivalent response, under TM‐polarised illumination (dashed).

Upon a phase transition, due to the higher refractive index and extinction coefficient of the PCM in its crystalline phase (Figure 2b), the spectral response of the metasurface after a non‐volatile phase transition is characterized by a red shifting of the designed reflection resonance alongside a lowering in quality factor translating to a period dependent tuning of the observed modulation contrast for reflection, transmission and absorption (**Figure** **5**a–c). We define the modulation contrast as the ratio between the amorphous and crystalline phase responses of the metasurface. Notably, due to the polarization‐sensitive nature of the nanograting metasurface employed here, a large polarization‐dependent modulation contrast can be engineered between the TE and TM polarized responses observed in the fabricated solution‐processed metasurfaces where modulation contrast swings as high as 2–3 dB is observed when the metasurface is illuminated with TE polarized light in contrast to ≈1dB observed for TM polarized incident light.

## 3 Conclusion

In conclusion, the solution‐processed films and metasurfaces presented here hold great promise in dramatically lowering the cost of production for a variety of smart/adaptive flat optic and electronic device platforms, taking advantage of PCM's as their programmable layer, easing their integration into various manufacturing lines by removing the need for the use of ultrahigh vacuum growth systems and/or cleanroom environments. To show their practical potential, we show that optical grade films can be synthesized, and fabrication of nanophotonic devices through common patterning techniques is perfectly possible through the demonstration of solution‐processed reconfigurable metasurfaces made from Sb_2_S_3_.

The significant optical contrast between phases, which is comparable to PVD films, and the associated low thermal drift and noise observed in the reduced thermo‐optic coefficients, when compared to their PVD counterparts across telecommunication frequencies, further emphasize their potential as a cost‐effective memory solution for zero‐static‐power, low noise/drift, non‐volatile signal modulation sought after in emerging metamaterial and photonic integrated circuit platforms. We also show that solution‐processed Sb_2_S_3_ exhibits broadband switchable thermo‐optic properties in the wavelength range λ < 1000nm, coinciding with much of the emission lines being explored from silicon, germanium, and nitrogen vacancies for integrated quantum photonic applications. Notably, the difficulties associated with integrating quantum emitters in the form of solution‐processed quantum dots and fluorescent nanodiamonds with pre‐grown PVD films are solved through the use of solution‐processed PCM's, where wet‐chemistry techniques can be used to synthesize hybrid mixtures, giving rise to a new generation of PCM‐emitter hybrid composites as integrated programmable single photon sources.

While focused ion beam (FIB) milling was used in this study to demonstrate the feasibility and performance of the reconfigurable metasurfaces, we recognize its inherent limitations in throughput and scalability. FIB is well‐suited for prototyping and high‐resolution patterning on small areas; however, for practical deployment and large‐area manufacturing, alternative scalable techniques need to be considered. Nanoimprint lithography (NIL) offers a promising solution, providing high‐resolution, large‐area replication of nanostructures with excellent fidelity and low cost per unit area. Interference lithography is another viable option, particularly suited for periodic metasurface designs, enabling wafer‐scale patterning without the need for physical masks. Both methods are compatible with the solution‐processed Sb₂S₃ films shown here and can support the fabrication on flexible or unconventional substrates.

Lastly, the liquid nature of the deposition technique lends itself perfectly for inclusion in display technologies as chalcogenides are also being explored globally for solid‐state flexible displays^[^ ^40^ ^]^; just as liquid crystals revolutionized the television industry (e.g LCD's), liquid chalcogenides can provide the same advantage to emerging display, telecommunication and computing architectures.

## Conflict of Interest

The authors declare no conflict of interest.

## Supporting information



Supporting Information

## Data Availability

The data that support the findings of this study are openly available in University of Alberta Dataverse Research Repository.
