# Supplementary material for: Low Noise and Drift Reconfigurable Solution‐Processed Chalcogenide Phase Change Metasurfaces
Source: Small Methods. 2025 Jul 24;10(2):e01088. doi: 10.1002/smtd.202501088 (PMC12825341; doi:10.1002/smtd.202501088)
Supplement: Supplementary file 1 — Supporting Information [file SMTD-10-e01088-s001.docx]

**Supporting information**

**Low noise and drift reconfigurable solution-processed chalcogenide phase change metasurfaces**

**Mahirah Zaini^1+^, Abbas Sheikh Ansari^1+^, Joshua Perkins^1^, Avik Mandal^1^, Yedeng Fei^1^, Ahmed H. Elfarash^1^, Tony Kong^1^ and Behrad Gholipour^1^**

*^1^Nanoscale Optics Lab, Electrical and Computer Engineering Dept, University of Alberta*

**Corresponding author e-mail:* [*bgholipo@ualberta.ca*](mailto:bgholipo@ualberta.ca)

*+These authors made an equal contribution.*

**Figure S1**


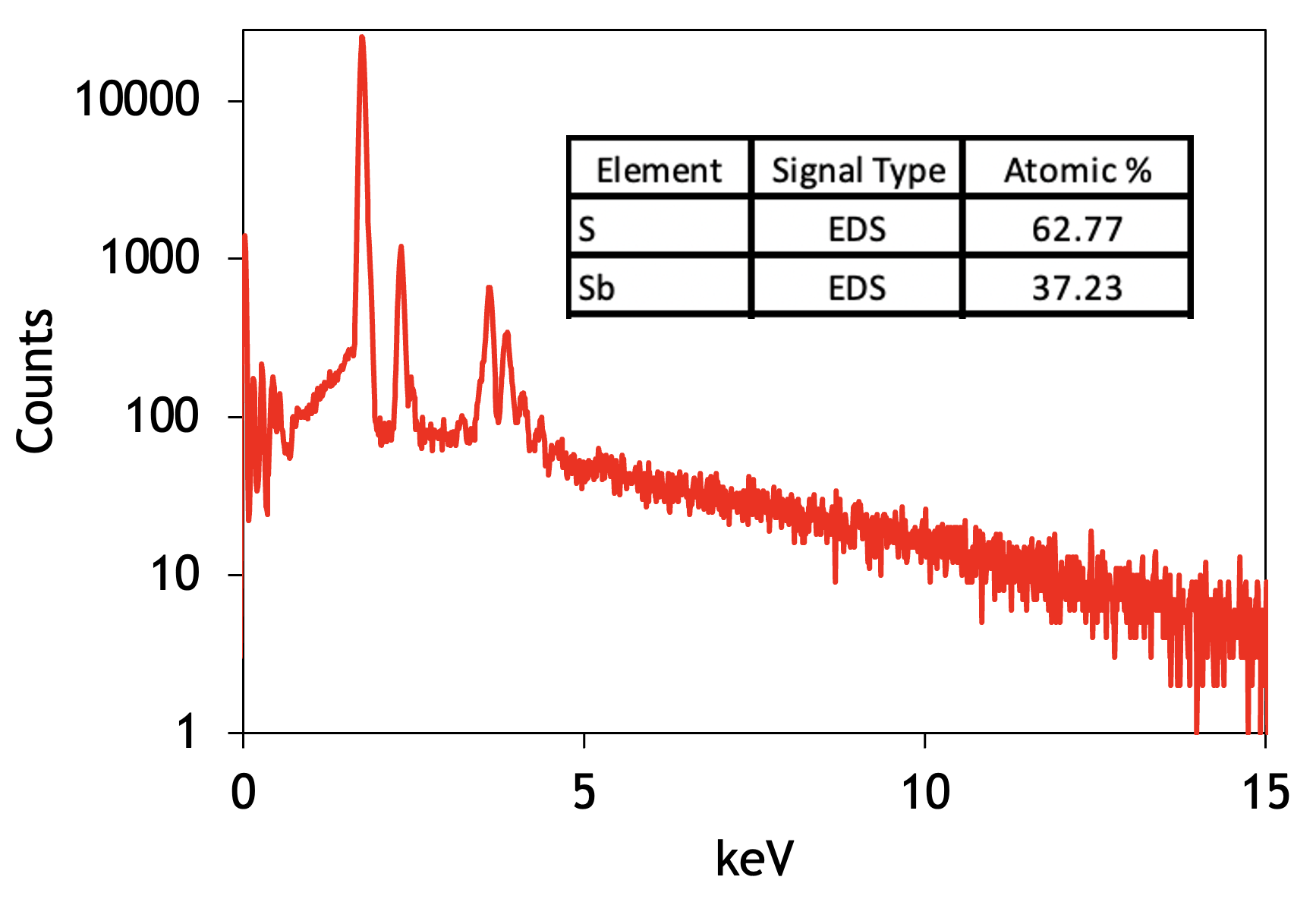


**Figure S1.** **Chemical compositional analysis of spin-coated solution-processed PCM.** Energy dispersive X-ray measurement of a typical **Sb_2_S_3_** film.

**Table S2**

| Demonstration | Modulation depth (Δ*n)* | Extinction Coefficient (k) Amorphous | Extinction Coefficient (k) Crystalline |
| --- | --- | --- | --- |
| Solution-processed Sb₂S₃ (This Work) | 1.2 | 0.3–0.5 (visible),  <0.1 (NIR) | 2.0–2.2 (visible), ~0.5–0.1 (NIR) |
| Teo et al., 2022^1^ | ~0.8 | negligible (<0.1 at 1550 nm) | lowest among PCMs tested (~0.1 at 1550 nm) |
| Delaney et al., 2020^2^ | 0.6 | <1e-5 at 1550 nm | <1e-5 at 1550 nm |
| Abdelraouf et al., 2023^3^ | 1.2 | ~0.4 (visible) | ~0.8 (visible) |

**Figure S3**


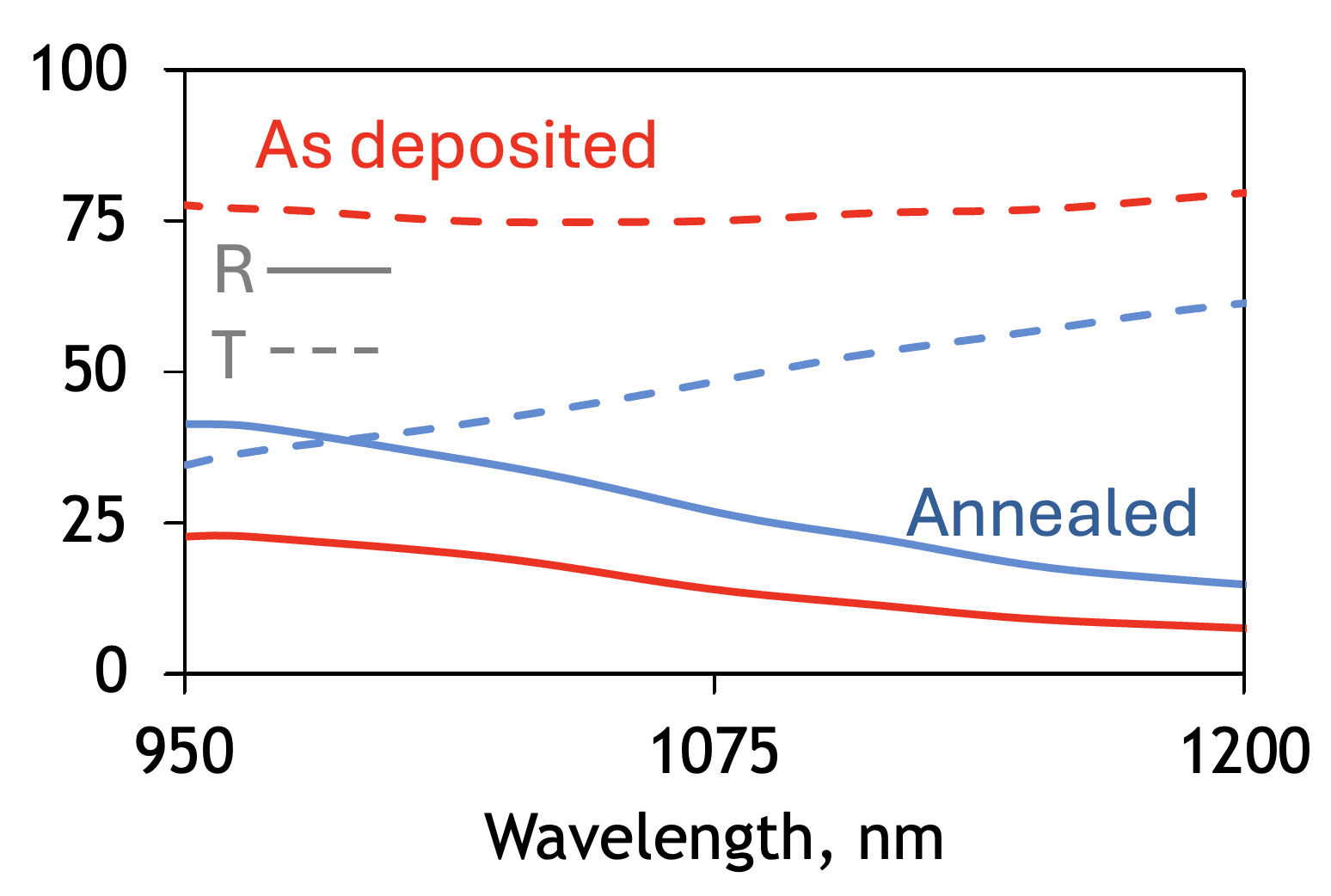


**Figure S3.** **Optical properties of an unstructured solution-processed PCM film.** Microspectrophotometrically measured reflection and transmission for a 380nm **Sb_2_S_3_** film.

**Figure S4**


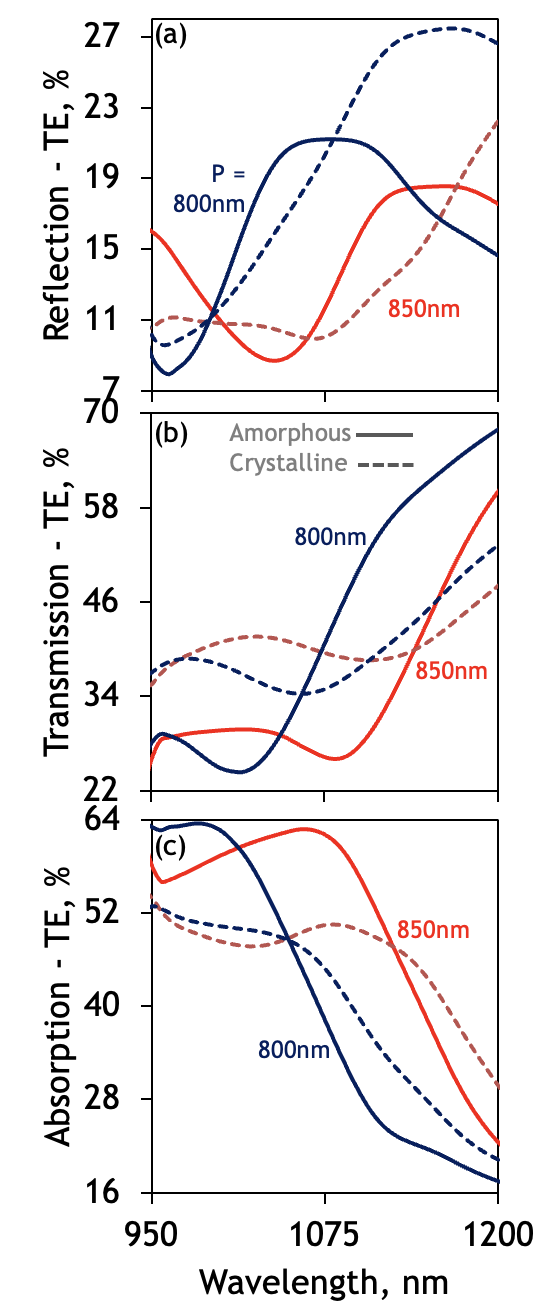


**Figure S4.** **Reconfigurable optical response of solution processed PCM metasurfaces.** Microspectrophotometrically measured reflection and transmission alongside absorption for metasurfaces with periods 800 and 850nm across both amorphous (solid) and crystalline (dashed) phases.

**References**

1. Teo, T. Y., Krbal, M., Mistrik, J., Prikryl, J., Lu, L., & Simpson, R. E. (2022). *Comparison and analysis of phase change materials-based reconfigurable silicon photonic directional couplers.* Optical Materials Express, 12(2), 606–621.
2. Delaney, M., Zeimpekis, I., Lawson, D., Hewak, D. W., & Muskens, O. L. (2020). *A New Family of Ultralow Loss Reversible Phase‐Change Materials for Photonic Integrated Circuits: Sb₂S₃ and Sb₂Se₃.* Advanced Functional Materials, 30(36), 2002447.
3. Abdelraouf, O. A. M., Wang, X. C., Goh Ken, C. H., Lim Nelson, C. B., Ng, S. K., Wang, W. D., Wang, X. R., Wang, Q. J., & Liu, H. (2023). *All‐Optical Switching of Structural Color with a Fabry‐Pérot Cavity.* Advanced Photonics Research, 4(11), 2300209.
